# Maternal pre-pregnancy risk drinking and toddler behavior problems: the Norwegian Mother and Child Cohort Study

**DOI:** 10.1007/s00787-014-0588-x

**Published:** 2014-07-23

**Authors:** Ann Kristin Knudsen, Jens Christoffer Skogen, Eivind Ystrom, Børge Sivertsen, Grethe S. Tell, Leila Torgersen

**Affiliations:** 1 Department of Global Public Health and Primary Care, University of Bergen, Bergen, Norway; 2 Department of Public Mental Health, Norwegian Institute of Public Health, Bergen, Norway; 3 Drug Research Western Norway, Stavanger University Hospital, Stavanger, Norway; 4 Department of Psychiatry, Helse Fonna HF, Haugesund, Norway; 5 Uni Health, Uni Research, Bergen, Norway; 6 Department of Genetics, Environment and Mental Health, Norwegian Institute of Public Health, Oslo, Norway; 7 Department of Psychosomatics and Health Behavior, Norwegian Institute of Public Health, Oslo, Norway

**Keywords:** Maternal risk drinking, Externalizing behavior problem, Internalizing behavior problems, T-ACE, Child Behavior Checklist, Cohort

## Abstract

Maternal risk drinking may be a risk factor for child behavior problems even if the mother has discontinued this behavior. Whether pre-pregnancy risk drinking is an independent predictor of child behavior problems, or whether a potential effect may be explained by maternal alcohol use during and after pregnancy or other adverse maternal characteristics, is not known. Employing data from the Norwegian Mother and Child Cohort Study (MoBa), longitudinal associations between maternal pre-pregnancy risk drinking and behavior problems in toddlers aged 18 and 36 months were examined. Included in the study was mothers answering MoBa questionnaires when the child was 18 (*N* = 56,682) and 36 months (*N* = 46,756), and who had responded to questions regarding pre-pregnancy risk drinking at gestation week 17/18, using the screening instrument T-ACE. Toddler behavior problems were measured with items from Child Behavior Checklist. Associations were analyzed with multivariate logistic regression, controlling for pre and postnatal alcohol use, as well as other relevant covariates. Pre-pregnancy risk drinking was associated with child behavior problems at 18 and 36 months, even after controlling for pre and postnatal alcohol use. Maternal ADHD and anxiety and depression were the only covariates that had any substantial impact on the associations. When all covariates were included in the model, the associations were weak for internalizing behavior problems and non-significant for externalizing behavior problems. Pre-pregnancy risk drinking may predict early development of behavior problems in the offspring. This increased risk may be due to other adverse maternal characteristics associated with risk drinking, in particular co-occurring maternal psychopathology.

## Introduction

Alcohol consumption among women of fertile age in northern Europe and the United States has increased dramatically in the last decades [1–3]. Increased alcohol consumption in the general female population is likely to lead to more women engaging in risk drinking [4]. Risk drinking may be defined as “drinking at levels or in patterns that increase the risk of alcohol-related harm” [5], that is: alcohol use that may indicate presence or risk of developing adverse physical, emotional and social outcomes [6]. Risk drinking may not only constitute a risk for the women herself but also to her children. Female risk drinking is particularly common in the fertile years [4], and women who engage in pre-pregnancy risk drinking are found to consume more alcohol both before and after being aware of the pregnancy compared with non-risk drinking women [7]. The adverse effect of prenatal alcohol exposure on offspring cognitive and behavioral outcomes is well known [8]. Most risk drinking women reduce or cease their alcohol consumption during pregnancy [7] and will hence in limited degree expose their fetuses for alcohol. However, as female risk drinking is associated with a range of other adverse outcomes, it may also in the absence of prenatal alcohol use confer a risk for later child maladjustment. For instance is female risk drinking, particularly in severer forms, associated with a disorganized and chaotic lifestyle, marital conflict, work disability, and psychopathology such as anxiety, depression and attention deficit hyperactivity disorder (ADHD) [7, 9–12]. Many risk drinking women also have risk drinking partners [7, 13]. Further, as alcohol use disorders include a genetic component, women engaging in risk drinking may also transfer genetic vulnerability to their children [14, 15]. Finally, one could speculate that some mothers engaging in pre-pregnancy risk drinking may return to risky drinking pattern after delivery and even more after they have stopped breastfeeding. Studies have found increased risk for maladjustment among older children of risk drinking mothers [16–18]. Thus, pre-pregnancy risk drinking behavior may be a predictor of child maladjustment independent of whether the mother ceases or continues her risk drinking behavior, through its association with other, potentially more pervasive risk factors for child maladjustment [11, 19, 20]. It is therefore necessary to control for such adverse factors before we can conclude that maternal pre-pregnancy risk drinking in itself is associated with child maladjustment.

Child maladjustment is commonly described in terms of internalizing and externalizing behavior problems [21, 22]. Internalizing behavior problems mainly affect the child’s internal psychological environment, and are characterized by anxious, withdrawn, inhibited or depressed behaviors and somatic complaints [21]. Externalizing behavior problems, in contrast, refer to outward directed behavior, in which the child is acting negatively on his or her surroundings [23]. Internalizing and externalizing behavior problems in toddlerhood are an important risk factor for subsequent school-age behavior problems [19]. For some, the problems will continue into adolescence and adulthood [19], resulting in anxiety and depressive disorders [22, 24], or school problems, juvenile delinquency, adult crime and violence, and adolescent substance abuse [23, 25]. Due to these potential catastrophic long-term consequences of toddler behavior problems, it is important to identify pertinent and preventable factors in the toddler environment associated with behavior problems.

Previous studies have found that school-age, adolescent and adult children of risk drinking mothers have increased levels of behavior problems [16–18]. However, although maternal risk drinking has been suggested to be an important predictor of offspring maladjustment, particularly in the early years of the child’s life [26], previous studies of very young children of risk drinking mothers have primarily focused on the effect of prenatal alcohol exposure. Increased levels of externalizing and internalizing behavior problems were found among preschool children in alcoholic families in a series of studies conducted by Edwards, Das Eiden and colleagues [27–29], but these studies mainly examined the impact of paternal risk drinking. Although a few of the children came from families where also the mother engaged in risk drinking after the child was born, the effect of maternal risk drinking could not be disentangled from co-occurring psychopathology and paternal risk drinking [27–29]. Families where the mother was considered likely to have consumed alcohol during pregnancy were excluded from these studies. A common weakness in the majority of other studies on children of risk drinking mothers is the lack of information on pre-pregnancy alcohol consumption [11, 30], precluding the interpretation of an effect of maternal risk drinking on child behavior problems beyond those caused by prenatal alcohol exposure [26].

It is thus an unanswered question whether pre-pregnancy risk drinking may predict toddler behavior problems, and whether this potential association is largely explained by maternal alcohol consumption during and/or after pregnancy or by other adverse factors known to be associated with maternal risk drinking. The aims of the present study were thus to (1) investigate whether maternal pre-pregnancy risk drinking predicted behavior problems in toddlers, and (2) whether a potential association could be explained by other risk factors associated with maternal risk drinking, including prenatal alcohol exposure, current maternal alcohol consumption, and maternal psychopathology.

## Methods

### Study design and participants

The Norwegian Mother and Child Cohort Study (MoBa) is an on-going prospective population-based pregnancy cohort study conducted by the Norwegian Institute of Public Health [31]. Participants were recruited between 1999 and 2008 and 277,700 pregnancies were invited. The participation rate was 40.6 %, and the cohort now includes over 109,000 children, 91,000 mothers, and 71,700 fathers. Non-participation was higher among pregnant women who were <25 years, living alone, smokers, and who had >2 previous births or previous stillbirths. MoBa mothers had somewhat higher education, a lower prevalence of preterm births and fewer babies with low birth weights compared with the general population of pregnant women in Norway in 1999 to 2008 [32]. Follow-up is still on-going through questionnaires at regular intervals and by linkage to national health registries. The study was approved by the Regional Committee for Medical Research Ethics in South-Eastern Norway, and has been performed in accordance with the ethical standards in the 1964 Declaration of Helsinki and its later amendments. Informed consent was obtained from each MoBa participant upon recruitment.

The current study employs information obtained at gestation week 17/18 and 30 (Questionnaire 1 (Q1) and 2 (Q2), and Questionnaire Father (QF)), and when the child was 6 months (Questionnaire 4; Q4), 18 months (Questionnaire 5; Q5), and 36 months old (Questionnaire 6; Q6). Additional information was obtained from the Medical Birth Registry of Norway (MBRN) which contains information about all births in Norway from 1967 and onwards [33]. Except from the MBRN and QF information, all information employed in the current study was obtained from mothers’ self-report.

**Fig. 1:**
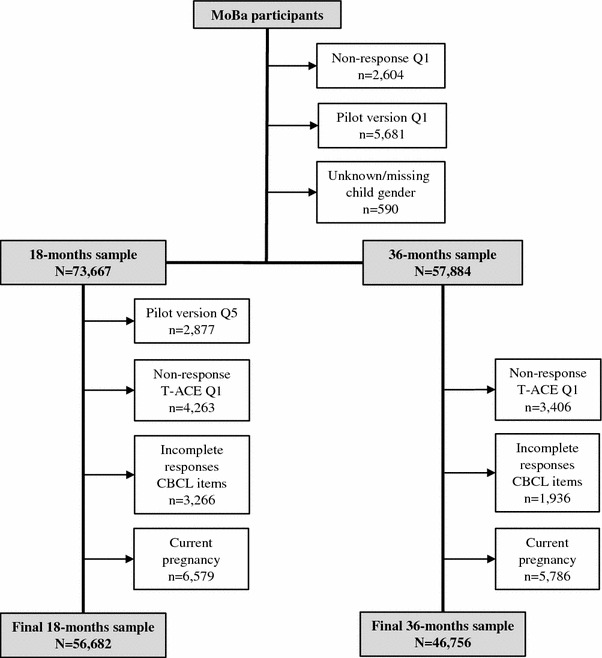
Flowchart detailing the exclusion processes leading to the 18 and 36 months samples included in the current study

The analyses in the current study are based on the two samples of participants who responded to the MoBa questionnaires when the child was 18 months (*n* = 56,682) and/or 36 months (*n* = 46,756). Mothers who answered items from the Child Behavior Checklist (CBCL) at 18 and/or 36 months, and responded to the T-ACE screening instrument in Q1, were included in the study. Mothers who had not answered T-ACE at Q1, who had incomplete responses to the CBCL items and who were currently pregnant were excluded from the samples. The exclusion processes are visualized in Fig. 1.

### Measures

Maternal pre-pregnancy risk drinking was assessed retrospectively with the screening instrument T-ACE at gestation week 17/18. T-ACE assesses risk for current drinking among pregnant women, but is also a sensitive screen for both general risk drinking and lifetime alcohol diagnoses [34, 35]. T-ACE consists of four items designed to measure tolerance to alcohol and behavior associated with risk drinking; Tolerance: “How many drinks does it take to make you feel high?”, Annoyed: “Have other people irritated you or hurt your feelings by criticizing how much you drink?”, Cut-down: “Have you ever felt that you ought to drink less alcohol?” and Eye-opener: “Have you ever drunk alcohol in the morning to calm your nerves or to get rid of a hangover?”. In MoBa, one alcohol unit was defined as equivalent to 1.5 cl (12.8 g) of pure alcohol. Although the conventional cut-off for the Tolerance item is two drinks, a cut-off of more than two drinks has been found to give higher specificity [36]. Further, risky single-occasion alcohol use among women is also usually defined as the intake of approximately 40–60 g pure alcohol (≈3.1–4.7 in MoBa alcohol units) or more in one single occasion [5, 37]. Thus, in the present study, two points were given if three or more drinks were reported in the first item (T). Affirmative responses on the A, C and E items were given one point each. A total score ≥3 was used as cut-off point for risk drinking, which has been shown to give good sensitivity and specificity [38, 39].

The Child Behavior Checklist [40] version for preschool children (CBCL/1.5–5) is constructed to cover a range of emotional, social and behavior problems. CBCL/1.5–5 consists of 99 items describing a behavior in the child the preceding 2 months, and the respondent, usually a parent or other adult who lives with the child, rates each item on a scale from 0 “not true”, 1 “somewhat or sometimes true” to 2 “very true or often true”. CBCL/1.5–5 has several subscales that are included in two scales measuring internalizing and externalizing behavior problems. The CBCL version for older children has been validated in a Norwegian study [41], while CBCL/1.5–5 has been validated in Dutch and Danish samples [42, 43]. MoBa was designed to cover a range of areas relevant for child health and development, and the full CBCL/1.5–5 was not included due to space restrictions in the questionnaires. The 18 and 36 months questionnaires in MoBa contain CBCL/1.5–5 items that were intended to represent all CBCL subscales with two or three items, and to be clinically and theoretically relevant indicators of behavior problems. Thirteen of the CBCL items were included in both the 18 and 36 months questionnaire, and were employed in the current study as indicators of behavior problems. The items are presented along with their internalizing or externalizing scale affiliation in Table 1. Case cut-points at the 85th or 90th percentile on each subscale are recommended when the full CBCL is employed in population-based studies [41, 42]. We adopted this strategy in the present study, thus scores above the 90th percentile were defined as internalizing or externalizing behavior problems.

**Table 1:** Items from the Child Behavior Checklist (CBCL) and their affiliated scale included in the 18 months (Q5) and 36 months (Q6) questionnaires in the MoBa Study

*Internalizing behavior problems*
Clings to adults or too dependent
Disturbed by any change in routine
Does not eat well
Gets too upset when separated from parents
Too fearful or anxious
*Externalizing behavior problems*
Can not concentrate, can not pay attention for long
Can not sit still restless or hyperactive
Quickly shift from one activity to another
Is defiant
Does not seem to feel guilty after misbehaving
Gets into many fights
Hits others
Punishment does not change his/her behavior

Information on child gender, birth weight and gestational age was obtained from MBRN and computed into a common* z*-scored variable indicating birth weight by gestational age. The number of older siblings was based on mothers’ report of previous pregnancies resulting in a live-born child in Q1. Maternal current civil status was assessed when the child was 18 and 36 months, and categorized as: (0) married/cohabitant, (1) divorced/separated, (2) widow, (3) single, and (4) other. Mother’s *highest completed education* was assessed at gestation week 17/18 and categorized as: (0) >4 years college/university, (1) ≤4 years college/university, (2) completed high school, (3) 1–2 years high school, and (4) 9 years compulsory school. Household income was based on maternal and paternal income if they were living together, reported at gestation week 17/18 and in QF. If the mother was not living with a spouse/partner, the household income was set equal to the mothers’ income. Six household income categories were generated, ranging from “No income” to “Over 1,000,000 NOK”. Finally, the mother was asked about recent financial problems at 18 and 36 months (yes/no).

The mother reported her alcohol consumption in each pregnancy trimester (Q1, Q3 and Q4) in terms of frequency: “How often do you consume alcohol?”, with alternatives ranging from “never” to “roughly 6–7 times a week”, quantity: “How many units do you usually drink when you consume alcohol?” with response categories ranging from “less than 1”, to “10 or more”, and binge drinking: “During pregnancy, how many times have you consumed five units or more of alcohol?”, with answer categories ranging from “never” to “several times per week”. The mother was also asked about her current frequency and quantity of alcohol intake when the child was 18 and 36 months old.

Mothers’ smoking habits during pregnancy were assessed in Q1, Q3 and Q4. Responses on these time points were computed into a common variable indicating smoking habits during the full pregnancy. The mother was coded as “daily smoker” if she reported to be smoking daily on either one of the assessments, “occasional smoker” if she reported occasional, and not daily smoking, on any of the assessments, and “never smoked” if she did not report daily or occasional smoking on any of the assessments.

Current maternal psychopathology included anxiety/depression assessed at 18 and 36 months and ADHD at 36 months. Maternal anxiety and depression were assessed with the eight items version of Hopkins Symptom CheckList (SCL-8) [44, 45]. The mothers were asked to indicate how much the presented emotions and cognitions had bothered them during the past 2 weeks, scored on a 4-point scale ranging from 1 (“not bothered”) to 4 (“very bothered”). The item scores were then summarized into a continuous variable, ranging from 8 to 32. Maternal ADHD was assessed in Q6 with the screening instrument Adult ADHD Self-Report Scale (ASRS) [46]. ASRS consists of six items measuring inattention and hyperactivity/impulsivity, with responses ranging from “never” to “very often”. The total scores of ASRS were summarized into a continuous variable, range 0–24, where higher scores indicate more ADHD symptoms.

### Statistical procedures

Missing responses in the included covariates ranged from 0.1 % (maternal age) to 29.2 % (maternal ADHD in the 18-months sample). Multiple missing imputation procedures in Stata 12.0, using the multivariate normal approach with 10 imputation procedures, were employed for these variables before the main analyses were conducted [47]. Attrition between Q1 and Q5/Q6 was somewhat higher among participants who scored at or above the cut-point on T-ACE at Q1 compared to participants scoring below the cut-point (attrition Q1–Q5 (cut-point vs. below cut-point): 31.1 vs. 26.2 % (*p* < 0.001), attrition Q1–Q6: 48.9 vs. 43.0 % (*p* < 0.001)). The characteristics of the two samples (18 and 36 months) were examined with descriptive statistics. The associations between maternal pre-pregnancy drinking problems and internalizing and externalizing behavior problems in their toddlers aged 18 and 36 months were analyzed using logistic regression models. The findings are presented as (1) crude estimates, (2) estimates adjusted for single covariates (except from the alcohol consumption variables, which were included as a common block at each trimester in pregnancy, and when the child was 18 and 36 months), and (3) fully adjusted estimates. All estimates are reported as odds ratios (OR) with 95 % confidence intervals (CI). Stata 12.0 was used for all analyses [48].

## Results

The characteristics of mothers and toddlers included in the 18 and 36 months samples are presented in Table 2. Pre-pregnancy risk drinking, defined as total T-ACE score ≥3, was reported by almost 4 % of the mothers in the 18 and 36 months samples.

**Table 2:** Characteristics of the MoBa participants when child is 18 and 36 months

Characteristics	18 months sample *N* = 56,682		36 months sample *N* = 46,756	
	*n* (%)	Mean (SD)	*n* (%)	Mean (SD)
Pre-pregnancy problem drinking (T-ACE)	2,206 (3.9)		1,769 (3.8)	
Internalizing behavior problems (CBCL)	3,106 (5.5)		4,008 (8.6)	
Externalizing behavior problems (CBCL)	3,969 (7.0)		4,002 (8.6)	
Child gender, boy	28,889 (51.0)		23,876 (51.1)	
Birth weight, gram		3,571 (582)		3,572 (584)
Gestation length, weeks		39.4 (1.9)		39.4 (1.9)
Maternal age, years^a^		30.5 (4.4)		30.6 (4.4)
Number of older siblings^a^		0.7 (0.5)		0.7 (0.8)
Current civil status, not married/cohabitant	2,350 (4.2)		2,429 (5.4)	
Highest completed education, <high school	3,074 (5.7)		2,346 (5.3)	
Household income before pregnancy, <300,000 NOK	6,137 (11.4)		4,995 (11.3)	
Recent financial problems	10,248(18.7)		5,446 (12.9)	
Alcohol consumption in pregnancy, ≥once a week^b^				
Week 0–12	1,621 (2.9)		1,321 (2.9)	
Week 13–24	312 (0.6)		291 (0.7)	
Week 25+	366 (0.7)		345 (0.7)	
Current alcohol consumption, ≥once a week^b^	4,412 (8.0)		10,947 (24.5)	
Smoking during pregnancy, daily^c^	3,024 (5.3)		2,469 (5.3)	
Maternal anxiety or depression (SCL-8)		10.3 (3.0)		10.3 (3.2)
ADHD^d^ (ASRS)		6.5 (3.4)		6.6 (3.5)

^a^When the child is born

^b^Reporting of alcohol consumption is in the present table restricted to the frequency of alcohol consumption

^c^Reported daily smoking in any period during pregnancy

^d^Maternal ADHD screened when child is 36 months (Q6)

Tables 3 and 4 describe the crude association between maternal pre-pregnancy risk drinking and toddler behavior problems, and changes in the OR estimates when single covariates were included in the models. The last line in each table shows the associations in the fully adjusted models. Maternal pre-pregnancy risk drinking was significantly associated with toddler internalizing and externalizing behavior problems at both 18 and 36 months. The association between risk drinking and internalizing behavior problems seemed to be slightly stronger at 36 months (OR 1.70, 95 % CI 1.48–1.96) compared to 18 months age (OR 1.50, 95 % CI 1.27–1.75); however, the two samples had overlapping CIs. The association between risk drinking and externalizing behavior problems did not differ at 18 months (OR 1.57, 95 % CI 1.36–1.81) and 36 months (OR 1.59, 95 % CI 1.38–1.84). Both current maternal anxiety/depression, and a positive screen for maternal ADHD reduced the estimates substantially, while the other covariates had little impact on the strength of the associations. Controlling for pre and postnatal alcohol consumption reduced the estimates between pre-pregnancy risk drinking and externalizing behavior problems, but had no effect on the estimates for internalizing behavior problems. In the fully adjusted model, statistically significant associations were only found for internalizing behavior problems at 36 months.

**Table 3:** Logistic regression analyses of maternal pre-pregnancy problem drinking and internalizing behavior problems in children aged 18 and 36 months, adjusted for potential confounders

Adjustment levels	18 months (*N* = 56,682)		36 months (*N* = 46,759)	
	OR	95 % CI	OR	95 % CI
Crude estimate	1.50***	1.27–1.75	1.70***	1.48–1.96
Child gender (reference: boy)	1.50***	1.27–1.75	1.70***	1.48–1.96
Birthweight by gestational age	1.48***	1.26–1.74	1.68***	1.46–1.93
Maternal age at birth	1.44***	1.22–1.69	1.64***	1.43–1.89
Number of older siblings	1.47***	1.25–1.72	1.56***	1.35–1.80
Maternal civil status (reference: married)	1.42***	1.21–1.67	1.61***	1.39–1.85
Completed education^a^ (reference ≥4 years university)	1.40***	1.19–1.64	1.61***	1.40–1.85
Household income^a^	1.42***	1.21–1.67	1.63***	1.42–1.88
Recent financial problems	1.38***	1.17–1.62	1.58***	1.37–1.82
Alcohol consumption in pregnancy^b^				
Week 0–12	1.48***	1.26–1.74	1.66***	1.44–1.92
Week 13–24	1.49***	1.27–1.75	1.71***	1.49–1.97
Week 25+	1.50***	1.28–1.76	1.72***	1.49–1.98
Smoking during pregnancy	1.42***	1.21–1.67	1.62***	1.40–1.86
Current alcohol consumption^c^	1.58***	1.34–1.85	1.72***	1.49–1.99
Maternal anxiety/depression	1.26**	1.07–1.48	1.45***	1.25–1.67
Maternal ADHD^d^	1.30**	1.10–1.52	1.42***	1.23–1.64
Adjusted for all covariates	1.17	0.98–1.38	1.23**	1.06–1.43

^a^Maternal completed education and household income measured at Q1

^b^Frequency, quantity and binge drinking

^c^Frequency and quantity

^d^Maternal ADHD screened when child is 36 months (Q6)

*p < 0.05; **p < 0.01; ***p < 0.001

**Table 4:** Logistic regression analyses of maternal pre-pregnancy problem drinking and externalizing behavior problems in children aged 18 and 36 months, adjusted for potential confounders

Adjustment levels	18 months (*N* = 56,682)		36 months (*N* = 46,759)	
	OR	95 % CI	OR	95 % CI
Crude estimate	1.57***	1.36–1.81	1.59***	1.38–1.84
Child gender (reference: boy)	1.57***	1.36–1.81	1.59***	1.38–1.84
Birthweight by gestational age	1.57***	1.36–1.81	1.58***	1.37–1.83
Maternal age at birth	1.49***	1.29–1.72	1.53***	1.32–1.76
Number of older siblings	1.54***	1.34–1.78	1.52***	1.31–1.75
Maternal civil status (reference: married)	1.52***	1.32–1.76	1.55***	1.34–1.79
Completed education^a^ (reference ≥4 years university)	1.48***	1.29–1.71	1.50***	1.30–1.74
Household income^a^	1.51***	1.31–1.74	1.54***	1.33–1.78
Recent financial problems	1.47***	1.27–1.69	1.49***	1.29–1.72
Alcohol consumption in pregnancy^b^				
Week 0–12	1.48***	1.28–1.71	1.53***	1.32–1.77
Week 13–24	1.56***	1.35–1.79	1.59***	1.37–1.83
Week 25+	1.56***	1.35–1.79	1.60***	1.38–1.85
Smoking during pregnancy	1.49***	1.29–1.72	1.50***	1.30–1.74
Current alcohol consumption^c^	1.40***	1.21–1.62	1.47***	1.27–1.70
Maternal anxiety/depression	1.37***	1.18–1.58	1.40***	1.20–1.61
Maternal ADHD^d^	1.32***	1.14–1.52	1.31***	1.13–1.51
Adjusted for all covariates	1.05	0.90–1.22	1.11	0.95–1.29

^a^Maternal completed education and household income measured at Q1

^b^Frequency, quantity and binge drinking

^c^Frequency and quantity

^d^Maternal ADHD screened when child is 36 months (Q6)

*p < 0.05; **p < 0.01; ***p < 0.001

## Discussion

Based on a sample of mothers and children from the general population, we found that maternal pre-pregnancy risk drinking was associated with internalizing and externalizing behavior problems in toddlerhood. The estimated prospective associations could not be fully explained by any single factor previously known to influence the relationship between parental risk drinking and child behavior problems, including maternal prenatal and postnatal alcohol consumption. Controlling for maternal psychopathology reduced the strength of the associations, and when all covariates were included in the model, the association was weak between pre-pregnancy risk drinking and internalizing behavior problems, and non-significant for externalizing behavior problems.

While paternal risk drinking is found to be associated with both internalizing and externalizing behavior problems among children of preschool age [26–29], studies examining the impact of maternal risk drinking on toddler behavior have largely focused on the effect of prenatal alcohol exposure [8, 49], or toddler outcomes in families where both parents are problem drinkers [27–29]. To our knowledge, this is the first study to examine the impact of maternal risk drinking before pregnancy on toddler behavior problems, independent of prenatal alcohol exposure and current maternal alcohol consumption. Therefore, our results contribute importantly to the identification of modifiable risk factors present before pregnancy that may influence the child’s early development. Possible interventions in that regard are discussed further below.

The associations between pre-pregnancy risk drinking and toddler behavior problems were only slightly attenuated by controlling for prenatal alcohol exposure in our sample. In general, the mothers included in the present study reported very low levels of prenatal alcohol consumption, and this limited our ability to control for the potential influence from the full range of alcohol consumption on our associations of interest. Prenatal alcohol exposure has a range of harmful effects on placental function and development [50], and the negative consequences of moderate to heavy prenatal alcohol exposure on the fetus are well known [8]. The evidence is less consistent on whether low to moderate prenatal alcohol exposure also constitutes a risk [8]. In a series of studies recently published from the Danish National Birth Cohort, no increased risk was found for adverse neurodevelopmental outcomes among children of women who drank less than nine units per week during early pregnancy [49]. Our findings, based on low prenatal alcohol exposure, may be in line with this. Several studies have shown that T-ACE is effective in predicting prenatal alcohol consumption, but to the best of our knowledge, only one study has examined whether T-ACE also further predicts toddler outcomes, in that case neurobehavioral deficits (lower IQ) among children of a small sample of African–American women of lower socioeconomic status [39]. We are not aware of other studies that have controlled for the effect of prenatal alcohol exposure on psychosocial outcomes in very young children of risk drinking mothers.

It is likely that maternal risk drinking behavior involves risk factors for toddler behavior problems beyond those caused by mere pre and postnatal alcohol consumption. We found that the inclusion of all covariates almost eliminated the association between pre-pregnancy risk drinking and toddler behavior problems. This may be interpreted in line with models explaining adverse development in children as a result of exposure to multiple risk factors [51]. Such risk factors may include genetics, low socioeconomic status, a chaotic and disorganized lifestyle, paternal alcohol or drug use, adverse parenting styles, low-quality family environment, and co-occurring parental psychopathology [9–15]. Risk drinking and alcohol use disorders are core facets of a common genetic risk factor for externalizing mental disorders, and this genetic vulnerability may transfer from the mother to the child [14, 15]. A recent review of children-of-twins (COT) studies concluded that the effect of parental alcohol problems on child ADHD, conduct problems and general externalizing problems was likely to be genetic in nature, with limited support of a direct environmental effect [52]. In terms of offspring internalizing disorder, the evidence was too scarce to draw a conclusion but preliminary findings suggested the presence of both genetic and environmental effects [52]. However, although no support was found for a direct environmental effect on externalizing disorders in these studies, many of the children of risk drinking parents grow up in homes with both genetic and environmental risks. Adverse environmental factors may have a moderating effect on the genetic risks of externalizing behavior problems [53].

It has been argued that behavior problems in toddlers are signals of disturbances in the relationship with the primary caregiver, rather than markers of psychopathology in the toddler [54]. Maternal psychopathology appeared to be a particularly potent explanatory factor for the associations between pre-pregnancy risk drinking and toddler behavior problems. Maternal psychopathology is an independent and strong risk factor for child behavior problems [19, 55], and the risk for adverse child outcomes increases if both risk drinking and psychopathology are present in the mother [56]. The literature is inconclusive on whether the effect of parental risk drinking on child outcomes is largely explained by parental psychopathology, but evidence suggests that these are important co-occurring risk factors [56]. Both maternal risk drinking and psychopathology may impact the mothers’ attachment and behavior towards her toddler [55, 56]. In the present study, we were not able to examine whether maternal pre-pregnancy risk drinking was associated with parenting deficits or poorer attachment, and this should be a focus of future studies.

A potential source of residual confounding is paternal risk drinking. Women who engage in risk drinking often have partners with similar problems [13, 17, 27], and although the mother may reduce or cease her risk drinking behavior when she becomes pregnant, this may not apply to the father and therefore constitutes an independent risk factor for child behavior problems [11, 26–28].

Regardless of whether pre-pregnancy risk drinking is an independent risk factor, or part of a larger constellation of genetic and environmental characteristics increasing the risk for toddler behavior problems, the current study indicates that pre-pregnancy risk drinking should be taken seriously. The mother is in regular contact with the health services during pregnancy and infant/toddler years, and routine screening for maternal risk drinking, for instance with T-ACE, may help target intervention programs and provide early identification of women and children who may need particularly extensive follow-up or support. Continuity and acceleration in behavior problems from toddler years to childhood, adolescence and adulthood highlights that intervention towards children and their families who display signals of psychosocial difficulties should be done as early as possible to prevent further development of the problems [19].

Previous research on pre-pregnancy risk drinking has largely focused on the associated risk for prenatal alcohol consumption, with less attention given to other adverse factors associated with maternal risk drinking. This selective focus may perhaps give an impression that factors beyond prenatal alcohol consumption are of less importance for child development and adjustment. Our findings indicate that this is not the case. More studies are, however, needed to understand mechanisms from pre-pregnancy risk drinking to child maladjustment beyond prenatal alcohol exposure.

### Limitations

The results from the present study should be considered in light of several important limitations. Firstly, the relatively low participation rate in MoBa (40.6 %) and attrition from Q1 to Q6 are likely to introduce selection bias [57]. Participation in population-based health studies is lower among individuals with alcohol problems [58]. Although selection bias is likely to have a greater impact on prevalence estimates than on association estimates [31, 57, 58], we cannot fully exclude the possibility that the associations identified in the present study underestimate the true relationships in a general population of mothers and toddlers. Secondly, under-reporting of alcohol consumption is a general challenge in health studies [59] and is particularly common in studies based on pregnancy samples [60]. Although a substantial portion of the mothers reported alcohol use during pregnancy, with falling tendencies from around 30 % in the first trimester, to 20 % in the second trimester and 10 % in the third trimester (data not shown), these numbers are lower than prevalence rates of pregnancy alcohol use from a previous Norwegian study by Alvik and colleagues [60], and may indicate under-reporting. However, bias due to residual confounding will only be present if under-reporting was more frequent among risk drinking than non-risk drinking women, and under-reporting has previously not been found to be higher among women scoring positive on T-ACE [60]. Thirdly, MoBa did not include the full CBCL 1.5/5 (for an overview of the items included, see Table 1). While the items indicating externalizing behavior problems at 18 months have been used in a previous publication from the MoBa database [61] the use of these abbreviated, and not validated versions of the original scales may threaten the validity of the results. Fourthly, the toddlers’ behavior problems were reported by the mother. Mothers with psychopathology or who engage in risk drinking may be more prone to both detect and interpret their children’s behavior in a more negative direction [51]. Maternal reporting of toddler behavior problems may thus reflect characteristics of the mother rather than the toddler. In contrast, Norwegian parents are in general found to under-recognize and under-report behavior problems in their children [62]. In the present study, mothers with psychopathology or risk drinking may over-report while “healthy” mothers under-report their toddlers’ behavior problems, resulting in falsely large group differences. Finally, many terms are used in the literature to describe harmful alcohol use, for instance “hazardous alcohol use”, “problem drinking”, “alcohol problems” and “alcoholism”, often without defining inclusion and exclusion criteria for the employed term. The choice of the concept “risk drinking” in the present study was based on this term being commonly used in studies employing T-ACE, although it lacks a theoretical rationale.

To conclude, we found that maternal pre-pregnancy risk drinking predicted toddler behavior problems. The associations were affected by other factors known to have importance in the relationship between parental risk drinking and child behavior problems. It is therefore likely that maternal pre-pregnancy risk drinking is not an independent risk factor for child behavior problems, but rather represents a proxy measure of other adverse characteristics which may influence her child’s behavior. A central issue for future research should be to identify and investigate such potential characteristics and how they in combination with each other and with risk drinking lead to adverse child outcomes. Further, developmental pathways in toddlers of mothers engaging in pre-pregnancy risk drinking should be investigated.
